# Cultivation of Different Oyster Mushroom (*Pleurotus* species) on Coffee Waste and Determination of Their Relative Biological Efficiency and Pectinase Enzyme Production, Ethiopia

**DOI:** 10.1155/2022/5219939

**Published:** 2022-05-05

**Authors:** Guta Dissasa

**Affiliations:** Institute of Biotechnology, Addis Ababa University, Addis Ababa, Ethiopia

## Abstract

Cultivation of specialty mushrooms on lignocellulosic wastes represents one of the most economic and cost-effective organic recycling processes. Solid-state cultivation (SSC) was carried out to evaluate the feasibility of using coffee waste (husk and parchment) as substrate for cultivation of oyster mushroom (*Pleurotus* species). The periods for spawn running, pinhead and fruit body formation, number of flushes, yield, and biological efficiency of the four *Pleurotus* species (*P. citrinopileatus*, *P. eryngii*, *P. ostreatus*, and *P. sapidus*) grown on coffee husk and parchment were studied. The results revealed that the time for the first appearance of pinhead was shortest for *P. ostreatus* (20–21) days followed by *P. sapidus* (22–23) days on coffee husks, while *P. eryngii* and *P. citrinopileatus* required 26–27 days and 23–24 days, respectively, on the some substrate. All the four *Pleurotus* species recorded at least four flushes and three flushes on coffee husk and parchment, respectively; flush 1 gave the highest yield while flush 3 and 4 gave the lowest yield. The biological efficiency (B.E.) for *P.* c*itrinopileatus*, *P. eryngii*, *P. ostreatus* and *P. sapidus* obtained from fresh coffee husk was 26.54, 40.94, 60.33, and 55.72, respectively. Significant differences (*P* < 0.05) in yield and % B.E. of the four mushrooms species were recorded. The results also showed that the B.E. (61.92%) of *P. ostreatus* grown on composted coffee husk was insignificantly higher (*P* < 0.05) than that grown on noncomposted coffee husk (60.33). The yields of *P. sapidus* obtained from the two substrates were almost comparable with that of *P. ostreatus*. There was a significant difference at (*P* < 0.05) observed between noncomposted and composted coffee husk and coffee parchment as well as between coffee husk and coffee parchment on yield and biological efficiency (B.E.). Composted coffee waste is more efficient than noncomposted one. Pectinase enzymes productions by these mushrooms were also studied. They are known to produce extracellular enzymes, particularly pectinase, which contribute to the biochemical decomposition of pectin-rich lignocellulosic wastes biomass. Accordingly, *P. sapidus* showed more pectolytic activities followed by *P. ostreatus*. But the pectolytic activity showed by *P. eryngii* and *P. citrinopileatus* was relatively lower. The implications of this study are the feasibility of using composted coffee husks and coffee parchment with the supplementary substrate to cultivate very protein-rich mushrooms for food in solid-state cultivation (SSC) while at the same time promoting environmental sustainability.

## 1. Introduction

Oyster mushrooms (*Pleurotus* species) are a heterogeneous group of the most commonly cultivated mushrooms industrially occupying 3rd place in the world [1]. Its popularity has been increasing due to its ease of cultivation, high yield potential, and high medicinal and nutritional value [2]. They are cultivated on various waste products of human, agricultural, forestry, and industrial activities, and by utilizing these wastes, they prevent environmental and health hazards posed by indiscriminate damping of these waste materials [3, 4].

In the tropics and subtropics, a large volume of unused lignocellulosic by-products can be found which are left to rot in the field or are disposed off through burning [5]. Coffee waste is among these wastes in abundance in the tropics. It is rich in anti-nutritional/anti-physiological factors such as tannins, caffeine, and phenolic compounds which have a high capacity to bind protein, making them unavailable for the microorganism and feed supplement for cattle and other livestock [6]. Consequently, most of the husk remains unutilized which leads to environmental pollution. Degradations of the toxic constituents of coffee husk to low level open new avenues for their utilization [7–9]. Cultivation of mushroom on these by-products may be one of the solutions to transforming these inedible wastes into accepted edible biomass of high market value, and the spent substrates are used as animal feed supplement [3, 5].

The use of spent mushroom substrate (SMS) in new cultivation cycles has economic and environmental viability. When considering the application of the circular economy concept in the production of edible mushrooms, the reuse of the SMS within the same process is highly attractive because it allows a better use of the biomass and the energy involved in the process and, therefore, tends to improve energy efficiency and resource conservation [10]. Within the circular bioeconomy, effective mushroom utilization through the key utilization of resources is fundamental in terms of producing profitable bioproducts, feasible improvement, and maximizing biological and socioeconomic benefits. Commercial mushrooms are delivered on biowaste such as straw, saw tidy, and wood chips. As such, mushroom-forming fungi change over low-quality waste streams into high-quality food [11].

These were due to their ability to produce high level of extracellular enzymes particularly polysaccharidase (cellulases, hemicellulases, pectinase, and ligninase) enzymes capable of degrading complex organic materials. Pectinase are the group of extracellular enzymes, which cause degradation of pectin, that are chain molecules with a rhamnogalacturonan backbone, associated with other polymers and carbohydrates [12, 13]. Biological efficiency (yield potentials) of cultivated mushrooms depends on the types of substrate used, the types of species/isolates employed (genetic nature), spawn type, and prevailing mushroom growing conditions [14].

Mushroom cultivation is reported as an economically viable biotechnology process for the conversion of various lignocellulosic wastes among which coffee wastes were the major ones. However, various lignocellulosic wastes such as rice bran, wheat straw, pulp, corncobs, cocoa shell waste, cotton waste, spent grain, sawdust, maize husks, and cassava peelings were commonly used as substrates than coffee wastes for mushroom production. Hence, this work was carried out to evaluate the feasibility of using coffee waste as substrate for the cultivation of different oyster mushroom (*Pleurotus* species) by using the polythene bag method of cultivation to determine their relative biological efficiency and their variation in pectinase enzyme production. The purpose is also to optimize coffee waste as substrate for the cultivation of this oyster mushroom and pave way for effective utilization of coffee wastes and reduction of environmental pollution caused by these wastes.

## 2. Methods

### 2.1. The Study Areas

The study was conducted at Addis Ababa University, College of Natural and Computational Science, Faculty of Life Science, Department of Microbial, Cellular, and Molecular Biology.

### 2.2. Experimental Design

Cross-sectional study design was conducted to evaluate the visibility of using coffee wastes as a substrate for oyster mushroom cultivation. Four *Pleurotus* species (*P. citrinopileatus* 0091*, P. eryngii* 0091*, P. ostreatus* 0091, and *P. sapidus* 0091) were used in this experiment. Malt extract agar (MEA) medium was used to culture the different *Pleurotus* species which were used for spawn preparations. 80% composted and noncomposted coffee parchment and coffee husk supplemented with cow dung (18%) and gypsum (2%) on dry weight basis were used as substrates to cultivate the mushroom by using the polythene bag (40 × 60 cm) method of cultivation, and their relative biological efficiency and variation in pectinase enzyme production were determined. Four (4) polythene bags having 300 gm composted and noncomposted coffee parchment and 375 gm composted and noncomposted coffee husk per *Pleurotus* species were used separately. Wooden boxes of 1 × 0.5 m size were used separately for compost preparation.

### 2.3. Sample Collection

All the materials used to carry out the experiment (coffee parchment, cow dung, wheat bran, gypsum, coffee husk, mushroom/*Pleurotus* species (*P. citrinopileatus* 0091, *P. eryngii* 0091*, P. ostreatus* 0091, and *P. sapidus* 0091) were obtained from Mycology Laboratory, Department of Microbial, Cellular, and Molecular Biology with the exception of sorghum, which were bought from local market in Addis Ababa.

### 2.4. Cultivation of *Pleurotus* on Malt Extract Agar (MEA)

The mushroom species, pure cultures of *P. citrinopileatus* 0091*, P. eryngii* 0091*, P. ostreatus* 0091, and *P. sapidus*, were maintained on PDA (Oxoid) slant at 4°C which were obtained from Mycology Laboratory, Department of Microbial, Cellular, and Molecular Biology. Eighteen (18) grams of MEA were added to 350 ml distilled water in 1 liter flask and were placed on a Bunsen burner to dissolve agar. After autoclaved at 121°c for 15 minutes, it was dispended into 9 cm diameter Petri dish as 20 ml per Petri dish. These were inoculated with *Pleurotus* species culture mentioned above by using cork borer which has a size of 6 mm diameter and incubated at 25°c for twelve (12) days. Mycelia growth in terms of diameter on culture plate was measured using a ruler to compare their growth rate with one another [15].

### 2.5. Cultivation on Coffee Wastes

Coffee parchment and coffee husk were used as substrate with cow dung and gypsum as supplementary substrates. Coffee husks are the major solid residues from the handling and processing of coffee and are chemically composed of 19–26% cellulose, 20% lignin, 13% pectin, 58–85% carbohydrate, and 8–11% protein on dry basis [16]. Coffee parchment is characterized chemically by a high concentration of crude fiber (62.1%), cellulose (46.1%), and lignin (34.2%) [7]. Cow dung contains about 3% nitrogen, 2% phosphorous, and 1% potassium [17]. It is a supplementary substrate serving as the nitrogen source in mushroom cultivation as fertilizers [18]. Gypsum contributes to the ionic strength of substrate solution and functions as buffer to control the pH value in the substrate [19].

In order to prepare the substrates, about 80% of coffee parchment or coffee husk alone was soaked overnight for 12 hours in water. The excess water was drained off, and the moisture was adjusted to 55–60% [20]. Then, it was supplemented with 20% supplementary substrate (18% cow dung and 2% gypsum) on dry weight basis. These substrates were distributed equally (300 gm coffee parchment and 375 gm coffee husk per polythene bag) into polythene bag of 40 × 60 cm size and autoclaved at 121°c for 30 min. A total of four (4) polythene bags having 300 gm (total of 1.20 kg) of supplemented coffee parchment and 375 gm (total of 1.5 kg) of supplemented coffee husk were used for each species. After cooling, they were inoculated with the 20 days old spawn (one glass bottle per bag) and mixed thoroughly to facilitate rapid and uniform mycelia growth. The mouth of the bag was tied using cotton plug and thread, and holes were made over the polythene bags for aeration. Then, they were incubated in the greenhouse at 27 C, and mycelia development in terms of diameter in the polythene bags was observed and measured using a ruler to compare their growth rate with one another [15].

### 2.6. Spawn Preparation

In order to prepare spawn, about 6 kg of sorghum was soaked for 12 hours in water. The excess water was drained off and (2%) wheat bran (common supplement in spawn preparation) [21] and (1%) gypsum were added. The ingredients were thoroughly mixed; moisture was adjusted to 55–60% with some modification, and the determination of moisture contents was undertaken according to Fan et al. [20]. Then, the mixture was distributed equally into 500 ml bottle, at the rate of 400 gm supplemented seed per bottle for a total of 16 bottles and autoclaved at 121°c for 30 min. After cooling, each bottle was inoculated with 12-day-old *Pleurotus* species culture (one plate per one glass jar). After 20 days of incubation at 25°c (when the mixture was totally invaded by mycelium), the spawn was ready to be used for the inoculation of the solid substrate [20].

### 2.7. Detection of Pectinase Enzymes Production (Pectolytic Activity)

Pectolytic activity (Pectinase enzymes production) of the different *Pleurotus* species was undertaken in a minimal liquid medium containing pectin as the only carbon source. The basal medium contains per liter, pectin of apple (sigma product): 10 gm, (NH4)_2_SO4: 2 gm, KH_2_PO_4_: 4 gm, and Na_2_HPO_4_: 6 gm were prepared in four different 500 ml flasks each having 250 ml of these basal mediums. The pH was adjusted to 7 with 0.2 M NaOH and HCl [22]. The medium was autoclaved at 121°c for 15 min. Four pieces of the pure culture of *P. citrinopileatus* 0091*, P. eryngii* 0091*, P. ostreatus* 0091, and *P. sapidus* 0091 species cut by cork borer (6 mm diameters) were inoculated into these liquid mediums separately and incubated for two weeks at 28°c. Then, the mycelium grown in these liquid mediums was filtered from the culture medium by using filter paper and then transferred to small aluminum caps. The one producing more pectinase enzyme has the capacity to degrade the pectin and yield more mycelium. The filtered mycelium is then dried to constant weight at 60°c in the oven and then weighed on an analytical balance, and the weight was recorded to the nearest milligram [22].

### 2.8. Substrate Preparation and Evaluation

#### 2.8.1. Compost Preparation

In order to prepare composted substrate, 4.8 kg of coffee parchment and 6 kg of coffee husk were soaked overnight for 12 hours in water separately. The excess water was drained off, and the moisture was adjusted to 55–60% [20]. It was then supplemented with 20% supplementary substrate, cow dung (18%) and gypsum (2%), on dry weight basis. Then, the mixture was filled in wooden boxes of 1 m × 0.5 m size separately and incubated aerobically outside the house under the shade for eight (8) days [21]. The temperature of the substrates recorded during the process was 24.5 C.

After eight (8) days of composting, these substrates were distributed equally into polythene bags of 40 × 60 cm size and autoclaved at 121°c for 30 min. After cooling, they were inoculated with the spawn (one glass bottle per polythene bag) and mixed thoroughly to facilitate rapid and uniform mycelial growth. The mouths of the bags were tied using the cotton plug, and thread and holes were made over the polythene bags for aeration. Then, they were incubated in the greenhouse at 27°C, and mycelial development in the polythene bags was observed and recorded.

### 2.9. Measurement of Growth

The growth was measured qualitatively and quantitatively through:

- Colony diameter measuring on Petri dish
- Mycelial invasion (visual observation and record mycelia growth)
- Biological efficiency

#### 2.9.1. Biological Efficiency

Biological efficiency (BE) was calculated as follows Fan et al. [20]:

(1) $$\begin{array}{c} \text{BE}=\frac{\text{Fresh weight of the mushroom}\times 100}{\text{Dry weight of the substrate}}. \end{array}$$

#### 2.9.2. Data Analysis

The data on mycelia growth rate on the three types of media and spawn running days, primordial formation days, mushroom yield, and biological efficiency of the four *Pleurotus* species (*P. citrinopileatus* 0091, *P. eryngii* 0091, *P. ostreatus* 0091, and *P. sapidus* 0091) cultivated on the two kinds of substrates (coffee husk and parchment) were subjected to analyses of variance (one-way ANOVA) at the 5% level using the statistical software JMPIN version 5.0.1 (John's Macintosh Project). Analyses were performed for all data with triplicates for each and were reported as the mean ± SD. The yield performance of the four *Pleurotus* species on composted and noncomposted coffee waste was tested using *t*-test (JMPIN version 5.0.1) [23].

## 3. Results

### 3.1. Culture Production and Mycelia Growth Rate on MEA Agar Plate and CHs and CP Substrates

During the present investigation, three types of media (one agar medium (plate) and two coffee waste substrates (in polythene bag)) were used, and different results were obtained for the different *Pleurotus* species. For each species, the result was determined and presented in Table 1. The result revealed that *P. ostreatus* showed relatively maximum average mycelial growth rate per day in diameter followed by *P. sapidus* on coffee parchment with 10.25 mm/day and 8.14 mm/day, on the malt extract agar media with 9.68 mm/day and 8.00 mm/day, and on coffee husks with 9.00 mm/day and 6.50 mm/day, respectively (Table 1). The lowest mycelia growth rate in diameter was observed in *P. eryngii* on the three media used (Table 1).

### 3.2. Spawn Preparation

**Figure 1:**
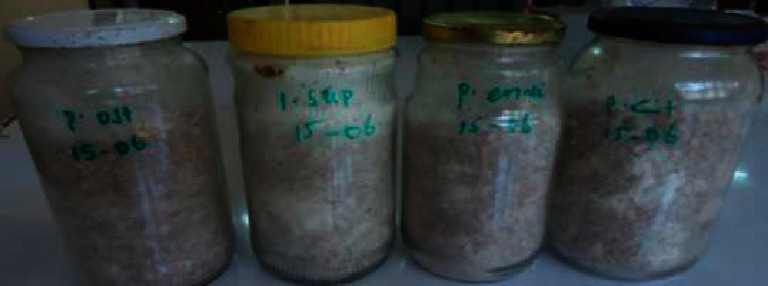
The four *Pleurotus* species spawn grown on sorghum after 20days of incubation.

Fully mycelial invasion of the four *Pleurotus* species (*P. citrinopileatus* 0091*, P. eryngii* 0091*, P. ostreatus* 0091, and *P. sapidus* 0091) was observed on sorghum after 20 days of incubation. It was ready to be used for the inoculation of the solid substrate (Figure 1).

### 3.3. Spawn Running (Mycelia Development) and Pin Head (Primordial) Formation of the Four Oyster Mushrooms

The two phases (spawn running and pinhead formation) which are important in the cultivation of any mushroom differed for the four *Pleurotus* species (oyster mushrooms) were investigated as shown in Table 2. It is evident from Table 2 that spawn running took two to three weeks after inoculation depending on the mushroom species and types of substrate used. *Pleurotus ostreatus* showed the shortest colonization time of 14 ± 1 days followed by *P. sapidus* at 15 ± 1 days on coffee parchment, whereas *P. ostreatus* showed a spawn run of 15 ± 1 days followed by *P. sapidus* at 16 ± 1 days for coffee husks while *P. eryngii* took relatively the longest colonization time of 20 ± 1 days followed by *P. citrinopileatus* at 17 ± 1 days on coffee husks.

Pinhead formation is the second stage of mycelia growth during the cultivation of mushrooms next to spawn running. Small pinhead-like structures were observed. As shown in Table 2, the time taken for these pinheads to be formed after the spawn running differed for each of the four mushrooms just like spawn running. *Pleurotus ostreatus* recorded the earliest pinhead formation, followed by *P. sapidus* and lastly *P. citrinopileatus* and *P. eryngii*, respectively. For *P. citrinopileatus, P. eryngii, P. ostreatus*, and *P. sapidus*, the time taken for the small pinhead to be formed was 23–24, 26–27, 20–21, and 22–23 days on coffee husks and 25–26, 28–29, 22–23, and 23–24 days on coffee parchment starting from the first day of spawning, respectively (Table 2).

For *P. ostreatus*, the time taken for the small pinhead to be formed on coffee husks was 20–21 days starting from the first day of spawning. These pinheads grew into mushrooms which were harvested 7–8 days later when young, firm, and fleshy. The length of the fruiting period was between 21–28 days. The entire crop cycle took about four weeks. Mushroom yields of the first flushes were harvested after two weeks of fructifications/six weeks of spawning (total of ≥40 days). These observations indicated that *P. ostreatus* has a short fruiting time; that is, in less than four weeks from the first day of spawning, mushroom biomass can be obtained followed by *P. sapidus*.

### 3.4. Mushroom Yield and Biological Efficiency on Coffee Parchment

The crop of four *Pleurotus* species mushrooms (12 weeks of cropping) was harvested for three flushes, and their mushroom yields on coffee parchment are given in Tables 3 and 4 below. The highest total weight of mushroom harvested on 1.20 kg dry substrate was recorded on composted coffee parchment for *P. ostreatus* 663.30 gm followed by *P. sapidus* 566.20 gm. *Pleurotus citrinopileatus* and *P. eryngii* yield 430.36 gm and 281.36 gm on the same substrate, respectively. While on noncomposted coffee parchment, they produce relatively minimum yield (Table 3).

The biological efficiency was worked out against the dry weight of noncomposted and composted coffee parchment and the fresh weight of mushrooms. It is clear from Tables 3 and 4 that the percentages of biological efficiency varied with mushroom species and substrate formulation. The fresh weight of mushrooms compared to the dry noncomposted coffee parchment on which they grew differed significantly between species and substrate formulation (*p* < 0.05). *Pleurotus ostreatus* gave the highest biological efficiency (B.E.) of (39.14%), followed by *P. sapidus* (32.74%)*. Pleurotus eryngii* and *P. citrinopileatus* gave a very low B.E. of 14.80% and 20.86%, respectively. In similar ways, *P. ostreatus* harvested from composted coffee parchment on a fresh weight basis gave the highest B.E. of 55.27% followed by *P. sapidus* (47.18%), *P. citrinopileatus* (35.86%), and *P. eryngii* (23.45%) (Table 4).

### 3.5. Mushroom Yield and Biological Efficiency on Coffee Husks

In general, cultivation on coffee husks provided higher yield and biological efficiency than coffee parchment. However, higher biological efficiency was obtained from composted coffee husk similar to coffee parchment (Table 5). The crop of four *Pleurotus* species mushrooms (12 weeks of cropping) was harvested for four flushes, and their mushroom yields on coffee husks are given in Tables 5 and 6. The highest total weight of mushroom harvested on 1.50 kg dry substrate was recorded on composted coffee husk for *P. ostreatus* (743.10 gm) followed by *P. sapidus* (697.96 gm). *Pleurotus citrinopileatus* and *P. eryngii* yield 496.68 gm and 361.20 gm on the same substrate, respectively (Table 5) while on noncomposted coffee husk, they produce relatively minimum yield just like coffee parchment (Table 6).

The biological efficiency was worked out against the dry weight of noncomposted and composted coffee husks and the fresh weight of mushrooms. It is clear from Tables 5 and 6 that the percentages of biological efficiency varied with mushroom species and substrate formulation. Accordingly, *P. ostreatus* gave the highest biological efficiency (61.92%) followed by *P. sapidus* (58.16%) on composted coffee husks. Relatively, *P. eryngii* and *P. citrinopileatus* produced lower biological efficiency on the same substrates having B.E. % of 30.10% and 41.39%, respectively (Table 5). Similarly, *P. ostreatus* harvested from noncomposted coffee husks on a fresh weight basis gave the highest B.E. of 60.33% followed by *P. sapidus* (55.72%), *P. citrinopileatus* (40.94%), and *P. eryngii* (27.74%), respectively (Table 6). When compared to composted coffee husks, yield and biological efficiency of the four *Pleurotus species* cultivated and harvested on 1.50 kg dry substrate of noncomposted coffee husks were relatively lower. Similarly, *P. ostreatus* harvested from noncomposted coffee husks on a fresh weight basis gave the highest B.E. of 57.46% followed by *P. sapidus* (49.67%). In all cases, fewer yield was produced by *P. eryngii* followed by *P. citrinopileatus* (Table 7).

### 3.6. Flushing Patterns of the Four *Pleurotus* Species on the Two Substrate

**Figure 2:**
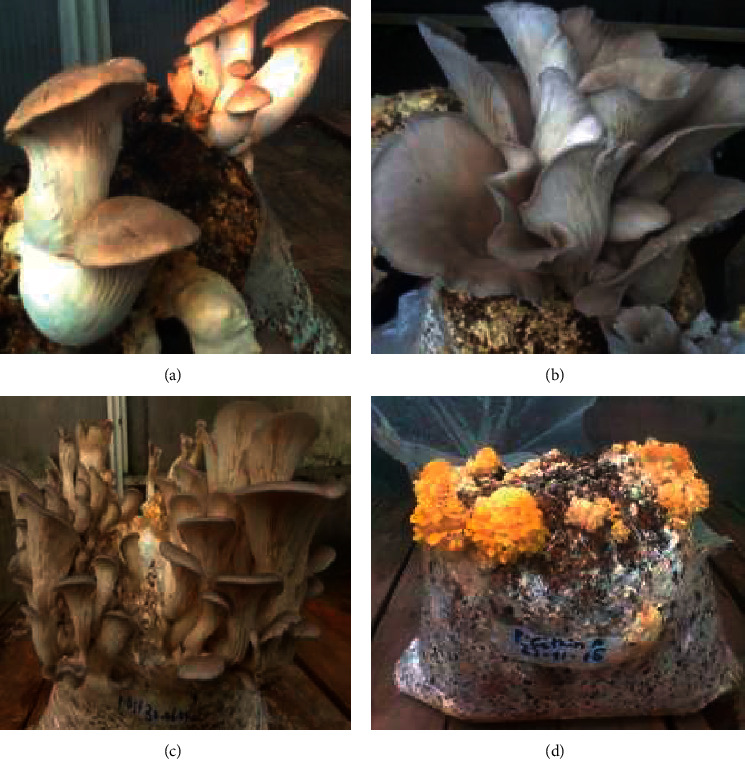
Fruiting bodies of the four *Pleurotus* species cultivated on coffee wastes (composted coffee husk) in the first flushes. A = *P. eryngii,* B *=* *P. sapidus*, C = *P. ostreatus*, and D = *P. citrinopileatus*.

Four flushes were recorded on composted and noncomposted coffee husks, whereas three flushes were recorded from composted and noncomposted coffee parchment. There was an expected progressive decline in yield over the course of the flushes from the four *Pleurotus* species cultivated on coffee parchment and husks, respectively (Tables 3–6). From the results, it seemed that substrates did not affect this pattern. Coffee husk is more efficient than coffee parchment by producing more flushes which corresponds to the production of much more yield and biological efficiency (Figure 2).

### 3.7. Detection of Pectinase Enzyme Production

Growth of the four *Pleurotus* species on liquid medium containing pectin as the only carbon source and revelation of growth and increase in mycelia biomass within two weeks of incubation demonstrated that the four species showed pectolytic activity (pectinase enzyme production). The results revealed that *P. sapidus* showed more pectolytic activities followed by *P. ostreatus* and *P. eryngii*, respectively (Table 8). But, a relatively lower pectolytic activity was seen in *P. citrinopileatus* (Table 8).

## 4. Discussion

In this study, the four *Pleurotus* species, *P. citrinopileatus, P. eryngii, P. ostreatus*, and *P. sapidus* showed different mycelia growth rate and colonization time on the different substrates used. It has been indicated that *P. ostreatus* recorded the shortest colonization time in the two substrate used followed by *P. sapidus*. However, longest mycelia colonization was seen in *P. eryngii* followed by *P. citrinopileatus.* Coffee parchment gave the fastest mycelia colonization time as compared to the mycelia colonization on coffee husks; however, this did not correspond with yield, indicating that mycelia colonization and yield of mushrooms have different requirements. This may be also due to variations in the structure, chemical composition, and nutrient content of the coffee parchment substrate. Statistically, there is a significant variation in their mycelia growth rate and mycelia colonization time on the growth substrates (*p* ≤ 0.05). This may be due to the difference in the type of substrate used and the type of species and/or the nature of strain employed. These results agree with the findings of Tekeste et al. [24] who showed the variations of mycelia colonization in oyster mushroom with the chemical composition of different substrates.

The time taken for pinheads (primordial) to be formed after the spawn running differed for each of the four mushrooms species. *Pleurotus ostreatus* recorded the earliest pinhead formation (20 days), followed by *P. sapidus* (22 days) and lastly *P. citrinopileatus* (23 days) and *P. eryngii* (26 days), respectively. This may be due to the variations in the extracellular enzyme production and the prevailing mushroom growing conditions since each *Pleurotus* species requires different environmental conditions of CO_2_ concentration, relative humidity, and temperature. These results are in agreement with the finding of Getachew et al. [25] who reported that *Pleurotus* species on different substrates took 2–4 weeks for fruiting bodies to be formed after inoculation of spawn, and Fan et al. [26] reported that the first fructification of *P. ostreatus* started 20 days after inoculation on coffee husk.

In the present study, the yield and biological efficiency of the four *Pleurotus* species, *P. citrinopileatus, P. eryngii, P. ostreatus*, and *P. sapidus*, cultivated on coffee husk and coffee parchment supplemented with the substrate (18% cow dung and 2% gypsum) were investigated. The results revealed that *P. ostreatus* yield the highest biological efficiency on the two substrate followed by *P. sapidus* while *P. eryngii* produce the least biological efficiency on the two substrates. The mushroom yields of *P. citrinopileatus, P. eryngii, P. ostreatus*, and *P. sapidus* obtained from composted coffee parchment were higher than those obtained from noncomposted coffee parchment by 15%, 8.65%, 16.13%, and 14.44% B.E., respectively. That is composted coffee parchment is more efficient than noncomposted coffee parchment by yielding relatively more biological efficiency. Similarly, the mushroom yields of *P. citrinopileatus, P. eryngii, P. ostreatus*, and *P. sapidus* obtained from composted coffee husks were higher than those obtained from noncomposted coffee husks by 1.19%, 3.56%, 1.59%, and 2.44% B.E., respectively. Shimelis [27] reported that composted coffee parchment and coffee husk produced more yield than noncomposted coffee parchment and coffee husk.

The overall B.E. % for composted coffee husks was inferior to the one recorded for *P. ostreatus* (96.5%) cultivated on coffee husk [26] and those recorded for *P. ostreatus* grown on the combination of cotton waste and coffee pulp which ranges from 52 to 79% [28]. However, a comparable B.E. of 53% was obtained from coffee wastes [29] and 64% was obtained from waste paper supplemented with cornstalk and wheat bran using *P. ostreatus* [30]. The B.E. 61.92% recorded for *P. ostreatus* from composted coffee husks is an indicative factor that composted substrates are more productive substrates in terms of bio efficiency as far as *Pleurotus* species are concerned. However, when the two substrates were compared (coffee husks and coffee parchment), coffee husks were more efficient than coffee parchment by producing on average a range of 6–10% more biological efficiency. This may be due to the variation in the nature of the two substrates (in their structures, chemical composition and C:N ratio). To this effect, a range of B.E. (60.33%–61.92%) was obtained from coffee husks for *P. ostreatus* followed by *P. sapidus* (55.72%–58.16%) while a range of B.E. 39.14%–55.27% and 32.74%–47.18% were obtained from coffee parchment for *P. ostreatus* and *P. sapidus*, respectively.

The four *Pleurotus* species did not yield high biological efficiency on noncomposted coffee wastes unlike the composted one. This may be due to the variations in the structure, chemical composition, and nutrient contents of composted and noncomposted coffee wastes. From the findings it can be concluded that using combined coffee parchment and coffee husk as substrate may produce more mushroom yield than using coffee parchment exclusively. This study showed the feasibility of using coffee wastes as substrates to cultivate oyster mushrooms. These results are in line with the finding of Fan et al. [26] and Tarko and Sirna [29] that showed the feasibility of coffee wastes as substrates to cultivate different *Pleurotus* species.

Approximately 91 to 96% of the total fresh weight was obtained in the first three consecutive flushes, with the last flushes producing 4 to 9% for the two substrates. These observations agree with those of Tsegaye and Tefera [28] who obtained 79% of the total fresh weight from the first three flushes and demonstrated that regardless of the mushroom species/strains and of the substrate (chemical and biological composition of the substrate) used to grow mushrooms, the pattern of gradually lessening mean yield per flush remains the same for cultivated oyster mushroom. Mshandete and Cuff [31] reported that flush gave more yield than the second and third flushes.

This has been attributed to the finding that the quantity of mushrooms harvested in each flush is directly proportional to the nutrients disappearing from the substrate. The assimilable nutrient sources (carbon and nitrogen) in the organic waste substrate were absorbed by mycelia translocated and mobilized to supply the fruit bodies [32]. It follows that in this study since the major part of mushroom production in the four *Pleurotus* species investigated was obtained in the first three flushes, the economic flushes could be limited to three flushes; the fourth flushes can be ignored. Shortening the cropping period by promoting rapid intensive early flushes could be of advantage in order to obtain maximum yield in a short time, which could ultimately lower the cost of production.

The cultivation in identical culture conditions of the four *Pleurotus* species revealed significant variations in their pectolytic activity (pectinase enzyme production) (*p* ≤ 0.05). Pectin is one of the carbon sources utilized by *Pleurotus* species and is suitable for mycelia growth [18]. The *Pleurotus* species able to produce more pectinase enzyme utilize the pectin in the medium easily and produce more mycelium biomass. The ability of mushroom species to degrade particular substrate depends on the production of enzymes necessary to degrade that substrate. Rajarathnam et al. [33] showed that although mushroom species have the ability to degrade lignocellulosic substrates, they exhibit differences regarding the production of enzymes necessary to degrade substrates and thus different abilities to grow to produce mycelium and fruit on residue substrates. This variation may be also due to the difference in the genetic nature of the particular *Pleurotus s*pecies/isolates employed, pH, and temperature at which they were incubated for enzyme production tests. This showed conformity with the finding of Atikpo et al. [34] who reported that the genetic nature of the mushroom species/strains determines their physiology and mycelia growth/colonization on different substrates.

## 5. Conclusion

The organic ingredients in coffee wastes were composted well within 8 days of composting, resulting in the formation of a suitable substrate for successful cultivation of *P. citrinopileatus, P. eryngii, P. ostreatus*, and *P. sapidus.* Composted coffee waste (coffee husk and parchment) as substrate for *Pleurotus* species proved to be a better substrate than noncomposted coffee waste in terms of mushroom productivity. The highest yield of each cultivated *Pleurotus* species was produced by *P. ostreatus* followed by *P. sapidus* on the two substrates used. The cultivation in identical culture conditions of the four *Pleurotus* species revealed significant variations in their pectolytic activity (*p* ≤ 0.05). This may be due to the difference in the genetic nature of the particular *Pleurotus s*pecies/isolates employed, pH, and temperature at which they were incubated for enzyme production tests.

## Figures and Tables

**Table 1:** Average mycelia growth rate per day in diameter (mm) of the four *Pleurotus* species on different media.

*Pleurotus* species	Culture media		
	CHs (substrate)	CP (substrate)	MEA (plate)
*P. citrinopileatus*	4.13 ± 0.10^a^	5.74 ± 0.26^a^	4.94 ± 0.25^a^
*P. eryngii*	4.00 ± 0.02^a^	5.31 ± 0.32^a^	4.71 ± 0.20^a^
*P. ostreatus*	9.00 ± 0.10^b^	10.25 ± 0.25^b^	9.68 ± 0.50^b^
*P. sapidus*	6.50 ± 0.50^c^	8.14 ± 0.50^c^	8.00 ± 0.50^c^

All values are means of triplicates ± SD. Levels not connected by the same letter under the same column are significantly different (*p* ≤ 0.05). SD = standard deviation, CH = coffee husk, CP = coffee parchment, MEA = malt extract agar.

**Table 2:** Spawn running (mycelia development) and primordial (pinhead) formation days of the four *Pleurotus* species on different substrates.

*Pleurotus* species	Substrate	Spawn running (days)	Pinhead formation (days)
*P. citrinopileatus*	CHs	18 ± 1^a^	23 ± 1^e^
	CP	17 ± 1^a^	25 ± 1^a^

*P. eryngii*	CHs	20 ± 1^b^	26 ± 1^a^
	CP	19 ± 1^b^	28 ± 1^b^

*P. ostreatus*	CHs	15 ± 1^c^	20 ± 1^g^
	CP	14 ± 1^c^	22 ± 1^h^

*P. sapidus*	CHs	17 ± 1^a^	22 ± 1^h^
	CP	16 ± 1^c^	23 ± 1^h^

All values are means of triplicates ± SD. Levels not connected by the same letter under the same column are significantly different (*p* ≤ 0.05). CHs = coffee husks, CP = coffee parchment, SD = standard deviation.

**Table 3:** Yield and biological efficiency of the four *Pleurotus* species on noncomposted coffee parchment substrate.

*Pleurotus species*	Flush (grams)				
	1^st^	2^nd^	3^rd^	Total	BE in%
*P. citrinopileatus*	126.34 ± 0.64^a^	84.36 ± 0.44^e^	39.65 ± 0.62^a^	250.36 ± 0.50^e^	20.86 ± 0.72^a^
*P. eryngii*	108.70 ± 0.60^b^	52.30 ± 0.54^f^	16.48 ± 0.51^b^	177.5 ± 0.31^f^	14.79 ± 0.79^b^
*P. ostreatus*	216.94 ± 0.77^c^	147.27 ± 0.51^g^	105.41 ± 0.42^c^	469.63 ± 0.76^g^	39.13 ± 0.52^c^
*P. sapidus*	210.81 ± 0.50^d^	127.47 ± 0.51^h^	54.68 ± 0.50^d^	392.96 ± 0.24^h^	32.74 ± 0.65^d^

All values are means of triplicates ± SD. Levels not connected by the same letter under the same column are significantly different (*p* ≤ 0.05).

**Table 4:** Yield and biological efficiency of the four *Pleurotus* species on composted coffee parchment substrate.

*Pleurotus species*	Flush (grams)				
	1^st^	2^nd^	3^rd^	Total	BE in%
*P. citrinopileatus*	228.00 ± 0.56^a^	157.00 ± 0.60^e^	45.36 ± 1.00^a^	430.40 ± 0.50^e^	35.86 ± 0.50^a^
*P. eryngii*	167.45 ± 0.78^b^	76.91 ± 0.42^f^	37.00 ± 0.48^b^	281.40 ± 0.50^f^	23.45 ± 0.79^b^
*P. ostreatus*	288.00 ± 0.53^c^	231.00 ± 0.52^g^	144.30 ± 0.72^c^	663.30 ± 0.51^g^	55.27 ± 0.63^c^
*P. sapidus*	264.81 ± 0.50^d^	194.73 ± 0.61^h^	106.64 ± 0.50^d^	566.20 ± 0.51^h^	47.18 ± 0.74^d^

All values are means of triplicates ± SD. Levels not connected by the same letter under the same column are significantly different (*p* ≤ 0.05).

**Table 5:** Yield and biological efficiency of the four *Pleurotus* species on composted coffee husks substrate.

*Pleurotus species*	Flush (grams)					
	1^st^	2^nd^	3^rd^	4^th^	Total	BE in %
*P. citrinopileatus*	281.00 ± 0.55^a^	145.50 ± 0.80^e^	58.43 ± 0.54^a^	11.75 ± 0.88^e^	496.68 ± 0.80^a^	41.39 ± 0.41^e^
*P. eryngii*	186.34 ± 0.50^b^	103.23 ± 0.91^f^	45.82 ± 0.80^b^	25.80 ± 0.79^f^	361.20 ± 0.96^b^	30.10 ± 0.52^f^
*P. ostreatus*	352.96 ± 0.64^c^	205.64 ± 0.32^g^	138.00 ± 0.85^c^	46.50 ± 0.36^g^	743.10 ± 0.68^c^	61.92 ± 0.37^g^
*P. sapidus*	318.44 ± 0.90^d^	202.68 ± 0.80^h^	164.70 ± 0.57^d^	57.27 ± 0.32^h^	697.96 ± 0.60^d^	58.16 ± 0.77^h^

All values are means of triplicates ± SD. Levels not connected by the same letter under the same column are significantly different (*p* ≤ 0.05).

**Table 6:** Yield and biological efficiency of the four *Pleurotus* species on composted coffee husks substrate.

*Pleurotus species*	Flush (grams)					
	1^st^	2^nd^	3^rd^	4^th^	Total	BE in%
*P. citrinopileatus*	286.36 ± 0.55^a^	102.72 ± 0.36^e^	66.22 ± 0.55^a^	36.00 ± 0.50^e^	491.32 ± 0.60^a^	40.94 ± 0.50^e^
*P. eryngii*	169.00 ± 0.63^b^	94.00 ± 0.51^f^	52.86 ± 0.51^b^	17.14 ± 0.60^f^	332.94 ± 0.57^b^	27.74 ± 0.40^f^
*P. ostreatus*	297.82 ± 0.54^c^	204.32 ± 0.53^g^	145.85 ± 0.61^c^	76.00 ± 0.13^g^	724.00 ± 0.74^c^	60.33 ± 0.58^g^
*P. sapidus*	293.80 ± 0.54^d^	197.34 ± 0.72^h^	130.53 ± 0.57^d^	47.00 ± 0.43^h^	668.67 ± 0.71^d^	55.72 ± 0.62^h^

All values are means of triplicates ± SD. Levels not connected by the same letter under the same column are significantly different (*p* < 0.05).

**Table 7:** Yield and biological efficiency of the four *Pleurotus* species on noncomposted coffee husks substrate.

*Pleurotus species*	Flush (grams)					
	1^st^	2^nd^	3^rd^	4^th^	Total	BE in %
*P. citrinopileatus*	294 ± 0.83^a^	180 ± 0.26^a^	76 ± 0.43^e^	NG	550 ± 0.56^e^	36.67 ± 0.57 ^a^
*P. eryngii*	248.50 ± 0.41^b^	152 ± 0.87 ^b^	58 ± 0.77^f^	NG	458.50 ± 0.76^f^	30.56 ± 0.46^b^
*P. ostreatus*	337 ± 0.53^c^	268 ± 0.84^c^	183 ± 0.34^g^	74 ± 0.37^a^	862 ± 0.46^g^	57.46 ± 0.41^c^
*P. sapidus*	318 ± 0.27^d^	207 ± 0.48^d^	134 ± 0.39^h^	86 ± 0.78^b^	745 ± 0.61^h^	49.67 ± 0.73^d^

All values are means of triplicates ± SD. Levels not connected by the same letter under the same column are significantly different (*p* < 0.05). NG = no growth.

**Table 8:** Mycelia dry weight of the four *Pleurotus* species grown on liquid medium containing pectin as the only carbon source for two weeks.

*Pleurotus species*	Mycelia dry weight (milligram)
*Pleurotus citrinopileatus*	130 ± 0.46^a^
*Pleurotus eryngii*	572 ± 0.81^c^
*Pleurotus ostreatus*	650 ± 0.73^b^
*Pleurotus sapidus*	784 ± 0.51^d^

All values are means of triplicates ± SD. Levels not connected by same letter under the same column are significantly different (*p* ≤ 0.05).

## Data Availability

All relevant data are within the paper.
